# Professional groups driving change toward patient-centred care: interprofessional working in stroke rehabilitation in Denmark

**DOI:** 10.1186/s12913-017-2603-7

**Published:** 2017-09-16

**Authors:** Viola Burau, Kathrine Carstensen, Stina Lou, Ellen Kuhlmann

**Affiliations:** 1 DEFACTUM – Public Health & Health Services Research, Aarhus, Central Denmark Region Denmark; 2 Department of Public Health, Aarhus University, Aarhus, Denmark; 3 Institute for Economics, Labour and Culture (IWAK), Goethe-University Frankfurt, Frankfurt, Germany; 4 Medical Management Centre, LIME, |Karolinska Institutet, Stockholm, Sweden

**Keywords:** Interprofessional working, Integrated care, Skill-mix, Health workforce governance, Professions driving organisational change, Patient-centred care, Stroke rehabilitation, Denmark

## Abstract

**Background:**

Patient-centred care based on needs has been gaining momentum in health policy and the workforce. This creates new demand for interprofessional teams and redefining roles and tasks of professionals, yet little is known on how to implement new health policies more effectively. Our aim was to analyse the role and capacity of health professions in driving organisational change in interprofessional working and patient-centred care.

**Methods:**

A case study of the introduction of interprofessional, early discharge teams in stroke rehabilitation in Denmark was conducted with focus on day-to-day coordination of care tasks and the professional groups’ interests and strategies. The study included 5 stroke teams and 17 interviews with different health professionals conducted in 2015.

**Results:**

Professional groups expressed highly positive professional interest in reorganised stroke rehabilitation concerning patients, professional practice and intersectoral relations; individual professional and collective interprofessional interests strongly coincided. The corresponding strategies were driven by a shared goal of providing needs-based care for patients. Individual professionals worked independently and on behalf of the team. There was also a degree of skills transfer as individual team members screened patients on behalf of other professional groups.

**Conclusions:**

The study identified supportive factors and contexts of patient-centred care. This highlights capacity to improve health workforce governance through professional participation, which should be explored more systematically in a wider range of healthcare services.

## Background

Internationally, there is growing agreement on the need for greater collaboration in healthcare, for example through interprofessional teams [1–3]. Interprofessional working is a response to demographic change and epidemiological transitions towards chronic illnesses and is a key element in policy reforms to improve primary healthcare and service provision based on the needs of patients [4–9]. This makes interprofessional working and the redefinition of roles and tasks of professionals an important issue of better health systems governance [10, 11], which requires greater attention from public health research and policy.

Available research has a number of limitations. It has primarily emphasised health professions as barriers to interprofessional working and delivery of needs-based care, and the negative effects of poor interprofessional collaboration on health services and patient care are well documented [4, 12–14]. Among the key factors making for poor collaboration, research has identified: power differentials, fragmented communication, lack of understanding of roles and different approaches to care [4, 12]. Moreover, an important underlying root cause is professional education [13, 15, 16]. In contrast, surprisingly little attention has been paid to the positive role of professionals as drivers of organisational change in interprofessional workplace settings.

The literature only reports few examples of organisational change driven by professionals for transformations in primary care [17], complementary and alternative medicine [18] or women-centred healthcare [19]. In all cases, diverse forms of integrating practice across professional boundaries and the emergence of new, joint professional practices occurred. This also reflects more integrated modes of professionalism that better connect professional and organisational change [20–22]. In all cases, health professionals have a shared experience that classic care provision based on a hierarchical division of labour and medical professions does not serve the needs of patients well. This recognition is a step towards a common goal to improve healthcare provision for patients. The relevance of shared interests and goals is confirmed by a recent, systematic literature review, which identified ‘the different actors' common interest in collaboration, perceiving opportunities to improve quality of care and to develop new professional fields’ as the ‘principal facilitator of interprofessional collaboration in primary care’ [4].

Combined, the findings suggest that, shared interests and goals are important facilitators of interprofessional working. In this context, stroke rehabilitation makes for an interesting case study for two reasons: it is strongly shaped by patients with multi-morbidity, who need complex care and services across the classic professional and sectoral boundaries; the commitment to teamwork seems to be stronger in stroke rehabilitation than in other areas [23, 24].

In this article, we place interprofessional working in the context of health policy and governance reform, using stroke rehabilitation in Denmark as a case study [25]. In 2012, Central Denmark Region carried out a major reorganisation of stroke treatment and rehabilitation. Previously, different hospitals in the region delivered acute stroke treatment and inpatient rehabilitation services. Following the reorganization, acute stroke treatment was centralised into two specialised regional hospitals and hospital stays were shortened. Stroke rehabilitation became the responsibility of newly established, interdisciplinary early discharge teams, one at each of the other 5 general regional hospitals. In each stroke team, 4 professional groups were represented: nurses, physiotherapists, occupational therapists and doctors. The team focuses on delivering individualised, initial stroke rehabilitation in the home of patients. In practice, this means the following: the stroke team visit patients 2–7 days after discharge to evaluate the patient’s needs and to draw up a rehabilitation plan; based on this, relevant members of the stroke team usually visit the patient’s home 1–4 times; and for those patients who need further professional rehabilitation the team makes a referral to relevant services provided by the respective home municipality and supports the transfer. The overall rationale underlying the establishment of the stroke teams thus was to ensure greater integration between hospital and community-based rehabilitation in the patients’ home municipality [25]. The region also introduced one regional network for all teams as well as 4 mono-professional, regional networks to support the implementation of the reorganisation. The idea was to strengthen peer support and stimulate organisational/professional development both among individual teams as new organisational entities and among members of the same professional group across teams [25]. The reorganisation of stroke treatment and rehabilitation reflects a health system that is highly integrated and where participatory modes of governance are strong, operating at different levels [26].

With our analysis of how health professionals engaged in the making of the new stroke teams, we hope to highlight ways to strengthen their participation in governance and thereby improve health system performance [11]. A better understanding of the capacity of professions to drive organisational change may promote more effective models of governing interprofessional working.

The study explores the following research questions:

- Which interests did the professional groups have in the introduction of interprofessional, early discharge teams?
- What specific strategies did the professional groups use in the process of establishing interprofessional working in stroke rehabilitation?

## Methods

We used a qualitative multiple-case design and included all 5 stroke teams established in 2012 following the reorganisation of stroke rehabilitation in Central Denmark Region. Our study focused on day-to-day coordination of care tasks in the stroke teams, also in relation to the municipalities, and the professional groups’ interests and strategies.

### Conceptual framework of the analysis

We combined approaches from the organisation and governance literatures on professions. This offers a multi-level perspective on professions by relating health care organisations to wider issues of the changing nature of steering health care systems. In the former field, a growing number of studies have turned their attention to the influence of professions on the creation, maintenance and transformation of organisational fields [22, 27, 28]. According to Suddaby and Viale, professionals use different strategies to influence organisational change. Governance studies of professions explore the role of professions as experts in changing public sector governance more broadly [29, 30]. Taken together, the research suggests that organisational change occurs in close interaction with professionals, and that health professionals are agents of organisational change in healthcare services and key players in strengthening governance.

The framework of the analysis consisted of two components: interests and strategies. The stroke team members belonged to distinct professional groups, and we expected each group to pursue its own professional interests. A relevant indicator was the extent to which professional groups saw the reorganisation as matching their specific professional interests in the development of stroke rehabilitation. Two strategies identified by Suddaby and Viale [27] seemed most relevant to our case. The first strategy, ‘Opening up new spaces of expertise’, concerned internally defining at-home stroke rehabilitation as a new practice area for the interprofessional stroke team. Expertise and knowledge increased with the daily organisation of the teamwork. The second strategy, ‘Populating existing organisational spaces with new actors’, aimed at creating a professional role for the new stroke team in community-based rehabilitation, specifically through organising collaboration with the care team in the municipalities.

### Data collection

Data were generated from semi-structured interviews with individual members of the stroke teams. The recruitment of informants aimed to include one member of each of the 4 professional groups represented in the stroke team: nurses, physiotherapists, occupational therapists and doctors. The stroke teams helped the researchers identify individual informants.

We thus intended to conduct 4 interviews in each of the 5 teams, but due to scheduling problems we had to skip 3 interviews. As the level of data saturation was satisfactory, we decided not to recruit additional participants. We conducted 17 individual interviews in total; interviewees had been employed between 1–8 years and were all female.

The interviews lasted 30–40 min and were conducted in person in autumn 2015 by a research assistant under close supervision of the senior members of the research team. She was a final year MA student and was highly familiar with the project through a previous 6-month internship. The interview guide was also tightly structured, following the conceptual framework outlined above and covered the following themes:

- professional interests in community-based stroke rehabilitation (Do the new teams make sense from the perspective of your professional practice, and what are the possibilities/challenges for improving the quality of stroke care?),
- professional strategies for division of labour within the teams (How does the team decide who should see the patient, how the patient should be treated and when the patient should be discharged?), and
- professional strategies for coordination with stroke rehabilitation in the municipalities (How does the team decide, who should coordinate patient treatment and discharge with the municipality, and how?).

### Data analysis

**Fig. 1:**
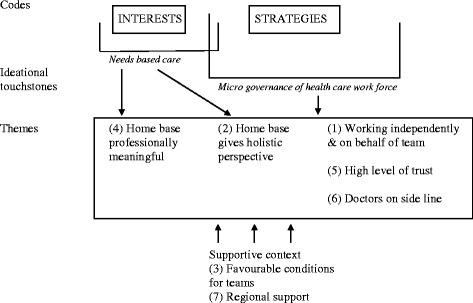
Overview of codes and themes

We started the analysis by constructing and applying a set of codes derived from the operationalisation of the conceptual framework. The first three authors independently performed preliminary coding of selected interviews based on three overall codes (professional interests, professional strategies for organising collaboration in the team, professional strategies for organising collaboration with the municipality). Following discussion among the authors the codes were refined and settled. Using NVivo 10 software we analysed the interview material based on a thematic approach that combined deductive and inductive elements to identify common threads [31]. The coded material was discussed among all authors and then collated to create preliminary themes, which were subsequently reviewed and refined (see Fig. 1 below). Within an overall logic of literal replication, we first conducted a within-case analysis, followed by a cross-case analysis and a search for check for any disconfirming evidence. We did this both individually and jointly, and the iterative nature of the work resulted in a truly joint analysis.

## Results

### Professional interests

Professional groups expressed highly positive interest in the reorganised stroke rehabilitation, and professional and collective interprofessional interests strongly coincided. The reorganisation offered services to a patient group that professionals felt had previously received sub-optimal rehabilitation services, as the following quote illustrates:

*‘[I]nitially, the interprofessional teams were a cost-cutting exercise to reduce the number of hospital beds. But in fact I feel that this is one of the few examples where cost cutting has helped to open new doors. I feel we have been able to target a patient group that was not included before.’* *Occupational therapist, Lakeside.*

The reorganisation had a very different initial rationale and this underlines the fact that professionals had a strong sense of meaningfulness. The same was true for patients, as a related study showed [32]. Meaningfulness centred on the notion of needs-based care, and professionals saw working in the patients’ homes as a way to achieve this. It gave them a more realistic and holistic impression as the following quote by a doctor demonstrates:

*‘[T]hey [patients] behave differently in their own homes; you can thus assess many cognitive issues. You can also easily smell if [the patient] is a smoker; you can see if this is the home of an addict. I get thousands of [snippets] of information, which I could not get at the hospital [...]. I feel this [working in the home] gives me a completely different impression.’* *Doctor, Valley.*

This suggests that working in the home gave professionals a more realistic and holistic impression and made it easier to spot other problems, and to assess the physical infrastructure of patients. Professionals were thus able to tailor treatment and training programmes to the specific needs and resources of individual patients. This also increased the quality of rehabilitation plans, as one physiotherapist stresses:

*‘[Professionally] I feel it makes a lot of sense that we come into people’s homes and complete our professional assessment [there]; we are as specific as possible when we draw up rehabilitation plans and pass them on to the professionals in the municipality. I feel our rehabilitation plans have become much better, because we use patients’ home environment to describe their needs [...].’* *Physiotherapist, Beachfront.*

Providing needs-based care also opened a temporal dimension with greater opportunities to follow patients in the different phases of their rehabilitation trajectory.

From the perspective of professionals, needs-based care had other positive consequences for their practice. This included greater autonomy as one nurse emphasised in the following quote.

*Researcher: ‘Does the way stroke rehabilitation is organized now give you greater influence over your work?’*

*Respondent: ‘Yes, definitely. [...]. I have more responsibility, because there are more issues where I have to find out if they require further [professional] attention.’* *Nurse, Mountainridge.*

Here greater autonomy was defined in terms of the number of issues that required independent decisions. Other respondents mentioned autonomy in terms of deciding how much time to spend with individual patients and designing individual treatment and training programmes. Professionals identified increased intersectoral coordination as another positive consequence; this was seen to prevent hospital readmissions and to reduce contact to elderly care services.

Working in a home setting was also challenging and required great flexibility as the professionals never knew what/whom they would meet. Other challenges related to the contents of professional practice. For example, the necessary screening on behalf of other professional groups (see below) can be time consuming and not always sufficient. A final set of challenges concerned the structural context of the stroke teams: Some professionals felt that hospital stays were sometimes too short, while existing resources put clear limits on the number of home visits and training available for patients in high need.

### Professional strategies: Organising collaboration in the team

The convergence of individual professional and collective professional interests was reflected in the professional strategies centring on needs-based care and (micro-level) health workforce governance. One team member of each team was responsible for assigning patients to other professionals in the team. All teams reported this process to be unproblematic. Professionals mainly drew on strategies of needs-based care: assignment was based on an assessment of patient needs and team resources, and one designated team member was responsible for the patient’s care trajectory.

Seeing patients in their homes reinforced strategies based on needs. It was difficult to maintain a narrow focus on the individual professional speciality in the face of the patient’s individual situation. For example, a nurse might have blood pressure and medication review as main professional responsibility, but in the home other challenges, such as cognitive difficulties or residual, physical impairments, clearly presented themselves and required attention.

The strategy of needs-based care motivated the individual professional to draw on the expertise of other professional groups as the following quote by a nurse illustrates:

*‘It is an all-round-picture [I get], because I have to be highly alert [to many issues]. If I am the first to visit [the patient], I check if there is anything cognitive and report [any problems] back to the other professionals. Or I quickly examine if there is anything physical.’* *Nurse, Mountainridge.*

Being able to include the perspective of other professions rather than to focus exclusively on their own, was exactly what constituted a competent and valued member of the stroke team. This type of engagement in (micro-level) health workforce governance included a number of strategies. The individual professionals worked both independently and on behalf of the team when they were in the homes of stroke patients. It was their responsibility to make a holistic assessment of the patient, initiate rehabilitation and organise timely discharge and transfer to rehabilitation in the municipality. Awareness of own professional strengths and shortcomings, and thus knowing when to include expertise of other team professionals, was integral to this practice. One occupational therapist explained this approach as follows:

*‘I have a sense that I do not have to see all patients. I have to see those where it is relevant. I feel we [in the interprofessional team] trust each other, that we draw on our [respective] expertise where this is relevant.’* *Occupational therapist, Lakeside.*

Thus, there was a degree of skills transfer among team professionals in the sense that they screened patients on behalf of other professions and assessed if such expertise was needed. This is not without pre-requisites, but requires trust as well as experience, as one physiotherapist stresses:

*‘This [working in the interprofessional team] requires experience. [...] [Y]ou have to have worked in neurology for a number of years and you have to have a well-established professional background to be able to work with other professionals and retain your own expertise, and to know what you can offer.’* *Physiotherapist, Lakeside.*

Experience meant not only extensive professional knowledge but also a clear sense of one’s professional strengths; this offered a springboard for defining high specific relationships of collaboration.

Importantly, this two-fold professional strategy to strengthen needs-based care and to promote health workforce governance by organising professional skills in the team in a collaborative way was facilitated by a number of context factors. Needs-based care had a functional and financial imperative; the team simply did not have the resources to send more than one staff member to the patients’ home. Skills transfer was eased by a high level of trust and mutual recognition of expertise in the teams. Professionals expressed confidence in their colleagues’ assessments, decisions and administration of resources and they trusted that their professional perspectives and expertise would be consulted if and when relevant. On the side-line, doctors acted as ad-hoc experts and took charge of formal discharge from team services. They expressed high levels of confidence in the quality of services provided by the team, as the following quote illustrates:

*‘I rely on the assessments the occupational therapists and the nurses make in the patients’ homes, because they [occupational therapists and nurses] have a lot of experience in this area [stroke rehabilitation].’* *Doctor, Seaside.*

Finally, the region had introduced a range of support structures for the teams, including networks across different localities and mono-professional networks.

### Professional strategies: Organising collaboration with the municipality

The stroke teams acted as link between hospital and municipalities. Again, the professionals here drew on a two-fold strategy combining needs-based care and health workforce governance. The team member responsible for the individual patient was in charge of handing over the patient to rehabilitation in the municipality. If a municipality was not ready to offer relevant services, the team continued its services, representing a form of skills overlap. The team members highly valued this flexibility as it allowed them to maintain focus on patient needs. This illustrates how closely the two strategies were intertwined. The skills overlap was slightly contested: the teams recognised that patients’ homes were usually considered the municipalities’ turf, and some professionals expressed concern that the level of professional expertise was sometimes lower in the municipalities. The teams also regularly met with their counterparts in the municipalities:

*‘We meet every 3 months. Initially, we mainly talked about individual patients. Now we discuss structural issues, what works and what does not work.’* *Nurse, Lakeside.*

As the meetings evolved, they offered a springboard to normalise the skills overlaps between the teams and the municipalities. This was further helped by the fact that with time the team knew their specific counterparts in the different municipalities they collaborated with; when handing over, they frequently talked on the phone and occasionally arranged to meet at the patient’s home.

## Discussion

Our case study of a health policy reform of stroke rehabilitation in Denmark showed highly positive interests of professional groups in reorganised care delivery, and the interests related to patient needs, professional practice and intersectoral relations. Individual professionals used strategies of working both independently and on behalf of the team in the homes of stroke patients. There was also a degree of skills transfer as individual team members screened patients on behalf of other professional groups. From the perspective of the professionals, the convergence of professional and collective interprofessional interests improved needs-based care and collaboration in the team. This is very interesting considering the focus on barriers in the literature and raises questions about the factors supporting this innovative behaviour.

This also affected the process of defining and adapting collaborative practices, which the literature insists is central for creating and maintaining teams [33–35]. The prevailing assumption is that this process is about constructing and maintaining professional boundaries [15, 35] and about securing legitimacy for particular skills [36]. The novelty of our results is that professionals no longer solely refer to mono-professional expertise; they see a mix of skills and awareness of other health professionals’ contributions as key conditions for serving the needs of patients.

From the literature shared interests and goals emerged as important factors facilitating interprofessional working [4]. Through the emergence of a new inclusive professionalism accountable to the needs of patients, these changes become sustained and have the potential to radically transform health service delivery [20–22]. In our study and from the perspective of professionals, the specific patient needs and organisational settings in stroke rehabilitation strengthened both; the bonds with patients (through working in home settings), making health professionals more sensitive to needs-based care; and the bonds with other professional groups (through interprofessional teams), making professionals more supportive of each other’s expertise.

This powerfully underlines another argument in the literature, that context factors at the level of systems and organisations are important for how well teams function [35, 37, 38]. Context includes general factors, for example how stroke rehabilitation is organised and funded and how professional practice is regulated, but also more specific factors, for example how new teams were supported at the beginning [35]. In the present case, the region had established a number of overlapping networks to support development and implementation of the reform, which may have served as an important, positive context factor.

Finally, the governance literature highlights the importance of participation as a key dimension of effective governance [11]. Although the importance of health professions as the backbone of health systems is increasingly recognised [39–41], their role in and capacity to drive organisational change in interprofessional working has not been examined in detail. Our study provides in-depth information on *why* professionals may act as facilitators of interprofessional work and *how* this can be supported by organisational and institutional conditions. The overall context of a highly integrated health system with participatory modes of governance like in Denmark [26] may offer more spaces for professionals acting as agents of organisational change than in other systems. It may therefore be useful to explore also in other health system settings how health professionals can be involved in health workforce governance in ways that improve interprofessional working and needs-based care.

Finally, our study has a number of limitations. Although all teams were included, only one member of each professional group was interviewed. Additional individual interviews, focus groups with the entire team and participant observations might have revealed a broader range of views. It would also have been interesting to include similar stroke rehabilitation teams in other regions.

## Conclusions

Our study set out to explore the role and capacity of health professional groups to establish more integrated and patient-centred service provision based on needs, using stroke rehabilitation in Denmark as a case study. From our research professional groups emerged as key governance players driving interprofessional working, drawing on individual professional as well as collective interprofessional perspectives. Working in the homes of patients supported health professionals in this role by creating functional and financial imperatives for interprofessional working. The study highlighted practice opportunities for improving health workforce governance through professional participation, potentially across a wide range of healthcare services. The Danish case study of stroke rehabilitation teams is embedded in a health system with a long tradition of more integrated forms of service provision and governance. This may foster the capacity of professional groups to innovate stroke rehabilitation more than in other health systems. However, our findings are also supported by governance research that has more generally identified strong stakeholder involvement as a main tool to improve governance [11].
